# *Choniomyzon taiwanensis* n. sp. (Crustacea: Copepoda: Nicothoidae) Parasitic on the External Egg Mass of the Longlegged Spiny Lobster *Panulirus longipes* *longipes* (Crustacea: Decapoda: Palinuridae) from Taiwanese Waters

**DOI:** 10.3390/ani11082475

**Published:** 2021-08-23

**Authors:** Yu-Rong Cheng, Kaori Wakabayashi, Yen-Ju Pan

**Affiliations:** 1 Department of Fisheries Production and Management, National Kaohsiung University of Science and Technology, No. 142 Haijhuan Rd., Nanzih District, Kaohsiung City 811213, Taiwan; yrcheng@nkust.edu.tw; 2 Graduate School of Integrated Science for Life, Hiroshima University, 1-4-4 Kagamiyama, Higashi-Hiroshima, Hiroshima 739-8528, Japan; kaoriw@hiroshima-u.ac.jp; 3 Department of Aquaculture, National Taiwan Ocean University, Keelung City 202301, Taiwan; 4 Center of Excellence for the Oceans, National Taiwan Ocean University, Keelung City 202301, Taiwan

**Keywords:** parasitic copepod, morphology, new species, SEM

## Abstract

**Simple Summary:**

This study reports an undescribed species of the *Choniomyzon* copepod obtained from a longlegged spiny lobster *Panulirus longipes longipes* (Milne-Edwards, 1868), collected from Hualien Port, Eastern Taiwan. We illustrated morphological features of the specimen to determine its taxonomic identification. The new species reported here is the first record of *Choniomyzon* species from spiny lobster in Taiwanese waters.

**Abstract:**

*Choniomyzon taiwanensis* n. sp. is described based on specimens collected from examining external egg masses of spiny lobster *Panulirus longipes longipes* (Milne-Edwards, 1868), obtained from Hualien, Taiwan. The new species differs from its congeners in possessing the following characteristics: (1) small prosome (about 0.84 mm); (2) armature of antennule being 1, 1, 2, 2, 1, 1, 1, 2, 1, 1+1 (aesthetasc), 4, 6+1 (aesthetasc); (3) five-segmented antenna; (4) second segment of antenna bearing 1 inner seta; (5) two-segmented maxilla. Based on the evidence of distinctive morphological features and host preference, *Choniomyzon taiwanensis* n. sp. is a new species. Until now, four species of *Choniomyzon* have been known living on decapods, and the new species reported here is the first record of *Choniomyzon* species from spiny lobster in Taiwanese waters.

## 1. Introduction

Nicothoid copepods are small, highly specialized parasites that live on other crustaceans [1,2]. So far, over 136 species are known living on/in a wide range of crustacean hosts [3,4]. They are found on the body surface, gills, and egg clutches or marsupia of their hosts [2,3]. Those copepods living in the egg clutches are very similar to their host eggs both in size and in their globular body. It is believed that they are egg predators and probably have evolved a specialized egg-mimicking behavior [1,5,6]. For example, in order to avoid ejection by a host, *Choniomyzon inflatus* Wakabayashi, Otake, Tanaka & Nagasawa, 2013, simulates the external eggs of its host in its prosome shape and size, and its caudal rami are similar to its host’s egg attachment filaments [6].

*Choniomyzon* Pillai, 1962, is a small genus of siphonostomatoid copepod in the family Nicothoidae Dana, 1849, and only three species have been recognized [4]. They are as follows: *Choniomyzon panuliri* Pillai, 1962, parasitic on the abdomen of spiny lobsters, *Panulirus homarus* Linnaeus, 1758, and *Panulirus versicolor* Latreille, 1804; *Choniomyzon libiniae* Santos & Björnberg, 2004, on the external eggs of an epialtid crab, *Libinia spinosa* Guérin, 1832; and *C*. *inflatus* on the external egg masses of the smooth fan lobster, *Ibacus novemdentatus* Gibbes, 1850 (Table 1).

In the present study, we illustrated an undescribed species of the *Choniomyzon* obtained from a longlegged spiny lobster *P*. *longipes longipes* collected from Hualien Port, Eastern Taiwan. Our study illustrates morphological features of the specimen to determine its taxonomic identification.

## 2. Materials and Methods

### 2.1. Specimen Collection

An egg-bearing female longlegged spiny lobster *P*. *longipes longipes* was purchased from a commercial fisherman in June 2020 at Hualien Port, Eastern Taiwan. Morphometric features (lengths of total body, cephalothorax, and abdomen and widths of cephalothorax and abdomen) of the lobster were measured. The external egg mass of the lobster was examined for the presence of parasitic copepods, and the copepod specimens were picked up by using insect forceps. A total of 37 female copepods were collected from the lobster. One live non-ovigerous female and one live ovigerous female copepod were photographed on a Motic BA210 compound microscope (Motic, China). Subsequently, all the copepod specimens were preserved in 70% ethanol.

### 2.2. Morphological Studies

Morphological observations of parasitic copepods were performed as described by Humes and Gooding in 1964 [11] for the standard procedures of examining symbiotic copepods. Length and width of the copepods were measured from five individuals under a dissecting microscope (ZE61, Olympus, Japan). Before dissection, the copepod specimens were immersed in 85% lactic acid for at least 1–2 h, then were dissected on a wooden slide under the dissecting microscope. The removed body parts and appendages were examined under a compound microscope (BX-43, Olympus, Japan). All drawings were made under the optical microscope with the aid of a drawing tube.

Four copepod specimens were transferred to 4% buffered glutaraldehyde for further analysis in scanning electron microscope (SEM). The specimens were dehydrated by an ethanol gradient (70% → 85% → 95% → 100%) and transferred on aluminum stubs with a drop of hexamethyldisilazane (HMDS) for critical point drying. The stubs were sputter-coated with gold (E1010, Hitachi Ltd., Tokyo, Japan) and then observed using a Hitachi TM4800 SEM (Hitachi Ltd., Tokyo, Japan).

## 3. Results

### 3.1. Taxonomic Account

**Order Siphonostomatoida Burmeister, 1835**

**Family Nicothoidae Dana, 1849**

**Genus *Choniomyzon* Pillai, 1962**

***Choniomyzon taiwanensis* n. sp.**

**Figure 1:**
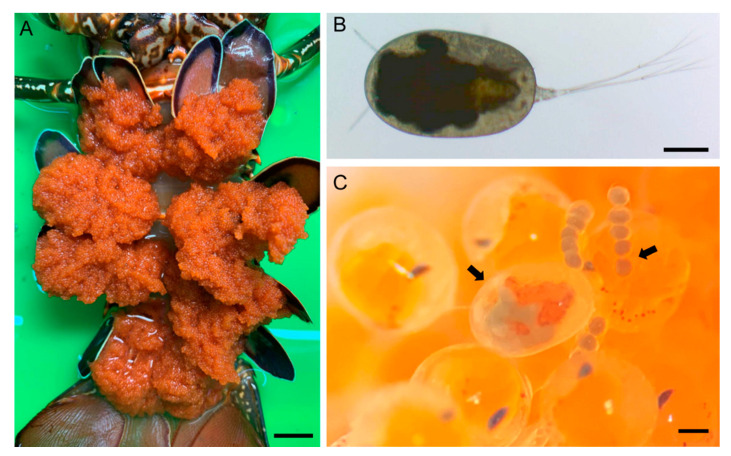
Living *Choniomyzon taiwanensis* n. sp. on the egg of host lobster *Panulirus longipes longipes*. (**A**) Ventral view (egg masses) of the *P*. *l. longipes* specimen, where the *C. taiwanensis* n. sp. specimens were collected; (**B**) female *C. taiwanensis*; (**C**) ovigerous female of *C. taiwanensis* (left arrow) carrying egg sac (right arrow) attached on the egg of *P. l. longipes*. Scale-bars: A, 2 cm; B and C, 200 µm.

**Figure 2:**
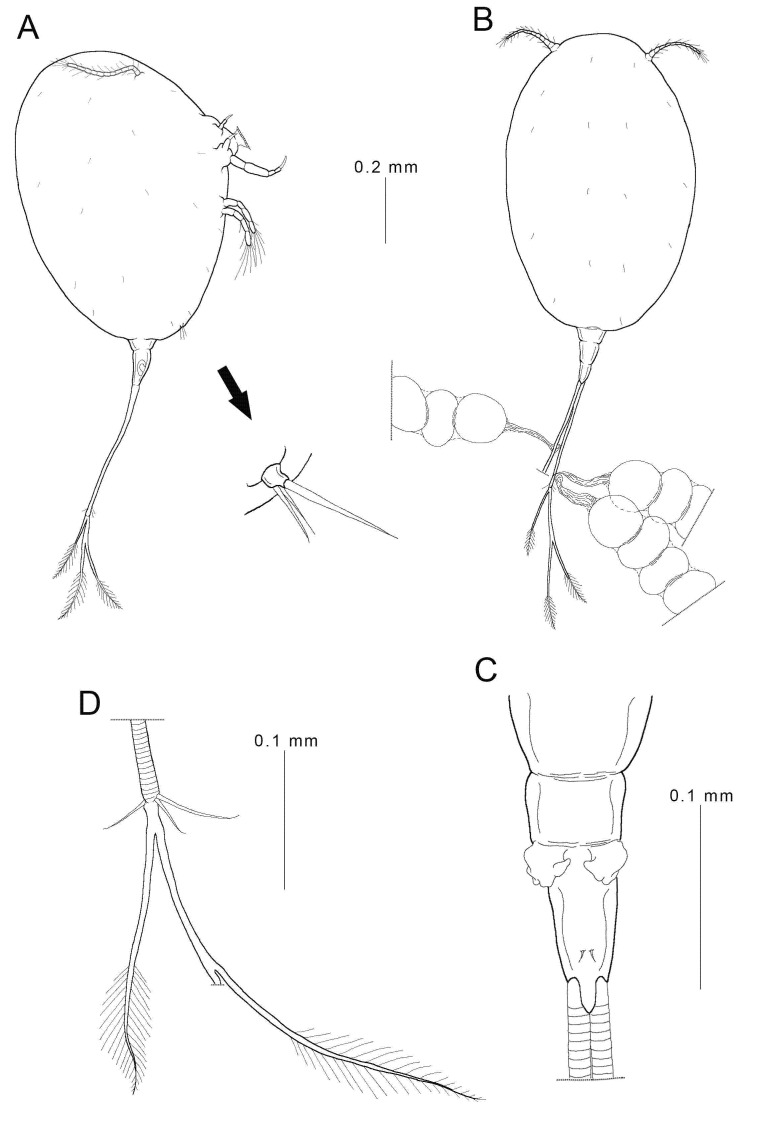
Female *Choniomyzon taiwanensis* n. sp. (**A**) Habitus, lateral (with enlarged leg 3); (**B**) female, habitus, dorsal (with three egg sacs); (**C**) urosome; (**D**) caudal ramus. Scale-bars: (**A**,**B**), 0.1 mm; (**C**,**D**), 0.02 mm.

**Figure 3:**
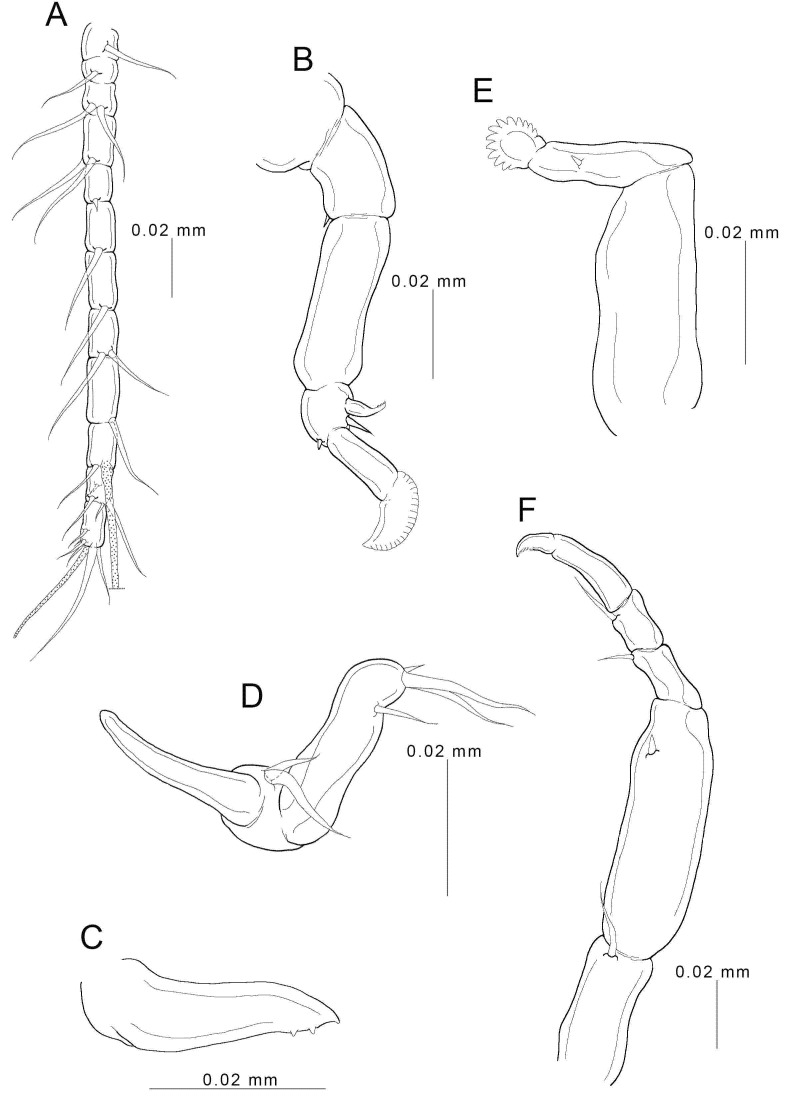
Female *Choniomyzon taiwanensis* n. sp. (**A**) Antennule; (**B**) antenna; (**C**) mandible; (**D**) maxillule; (**E**) maxilla; (**F**) maxilliped. Scale-bars: (**A**,**B**,**F**), 0.02 mm; (**C**–**E**), 0.01 mm.

**Figure 4:**
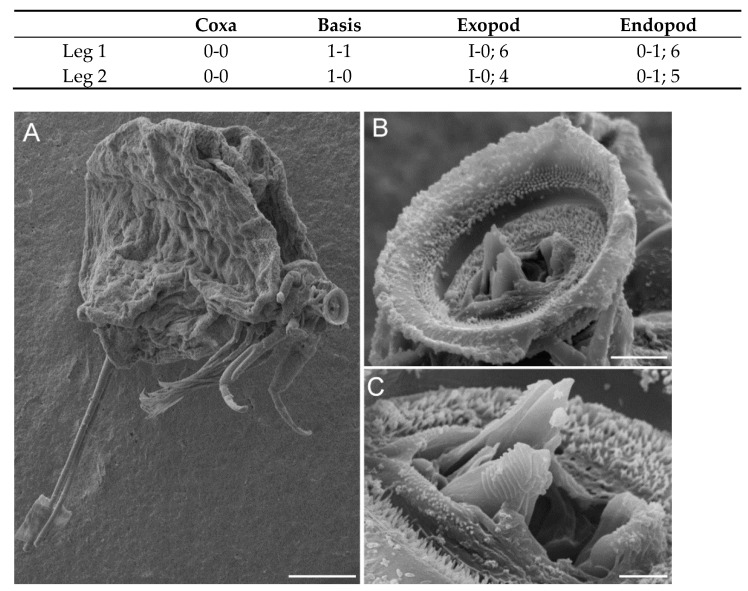
Scanning electric micrographs of *Choniomyzon taiwanensis* n. sp. (**A**) Adult female, right lateral; (**B**) oral region, postero-ventral; (**C**) tip of the mandible (MD) projects from the distal opening of oral cone (OC). Scale-bars: (**A**) 100 µm; (**B**) 10 µm; (**C**) 3 µm.

**Figure 5:**
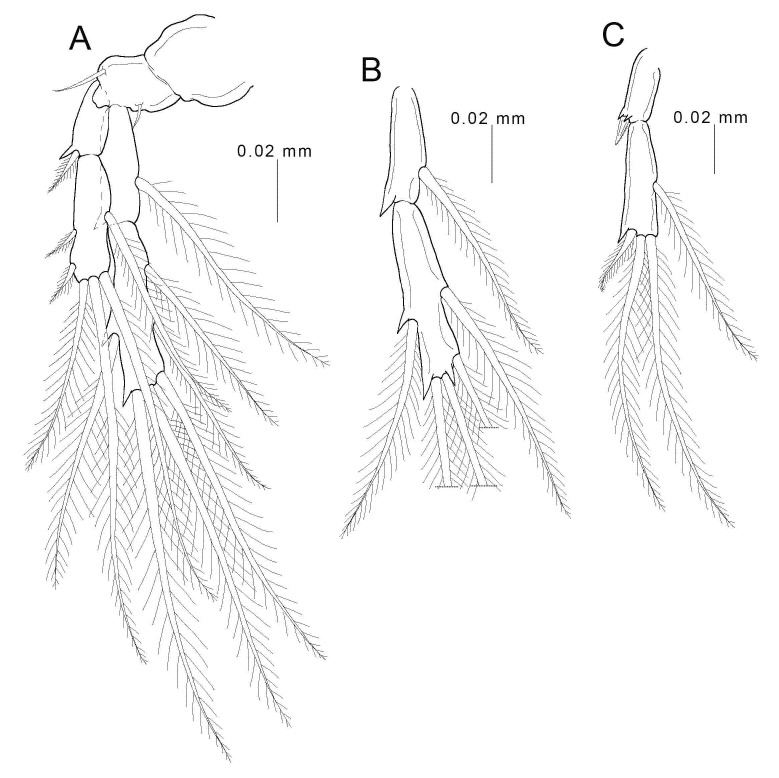
Female *Choniomyzon taiwanensis* n. sp. (**A**) Leg 1; (**B**) endopod of leg 2; (**C**) exopod of leg 2. Scale-bars: (**A**–**C**), 0.02 mm.

**Figure 1, Figure 2, Figure 3, Figure 4** and **Figure 5**

**Materials examined**

***Holotype****:* one ovigerous female (NTUM-Inv-10017). ***Paratype***: one ovigerous female (NTUM-Ivn-10018). Types were deposited in the National Taiwan University Museum (NTUM). ***Other materials***: 30 females, deposited in the Department of Fisheries Production and Management, National Kaohsiung University of Science and Technology, Kaohsiung, Taiwan.

***Type host*:***P*. *longipes longipes* (Crustacea: Decapoda: Achelata: Palinuridae). ***Morphometric measurement***: total body length: 193.84 mm, abdomen length: 108.13 mm, cephalothorax length: 85.71 mm, cephalothorax width: 50.46 mm, and abdomen width: 40.52 mm (Figure 1). ***Attachment site****:* external egg mass.

***Type locality:*** Hualien Port, Eastern Taiwan. The specimen was purchased from a commercial fisherman.

### 3.2. Description

**Female:** Body (Figure 1B,C and Figure 2A,B) oval-shaped, measuring 0.98 (0.82–1.04) mm in total length (without caudal ramus) and 0.80 (0.77–0.82) mm in greatest width, based on five specimens. Prosome (Figure 2A,B) ovoid, relatively large, 0.84 mm in length and 0.57 mm in width, ratio of prosome length to greatest width about 1.48:1; segmentation of prosome incomplete, formed by cephalothorax and three pedigerous somites. Urosome (Figure 2A–C) short, 0.14 mm in length and 0.07 mm in width, much shorter than the prosome and not being overlapped by the prosome; probably three-segmented; areas of attachment of egg sacs located dorso-laterally. Egg sac (Figure 2B) connected with caudal rami by membranous stalks; 6–10 eggs in each sac; up to three egg sacs per caudal ramus. Caudal ramus (Figure 2D) elongated, bearing three short, medial naked setae and three long, terminal plumose setae. Surface of body covered with denticles.

Antennule (Figure 3A) 12-segmented; armature: 1, 1, 2, 2, 1, 1, 1, 2, 1, 1+1 (aesthetasc), 4, 6+1 (aesthetasc); all setae naked. Antenna (Figure 3B) prehensile, four-segmented; basal segment bearing one small seta; second segment unarmed; third segment carrying one robust serrate spine (about one-half length of terminal segment), one seta, and one small spine; distal segment slender and bearing terminal serrate expansion. Mandible (Figure 3C and Figure 4C) stout, with pointed blade fringed with teeth on sub-terminal side, located within oral cone, only tip projecting from terminal side. Oral cone (Figure 4B,C) terminating in discoid sucker of about 30 µm in diameter; inner surface of sucker ornamented with pointed denticles. Maxillule (Figure 3D) bilobed; inner lobe tipped with cylindrical process and two basal, unequal setae; outer lobe cylindrical process bearing two long, terminal setae, one small seta, and one sub-terminal seta. Maxilla (Figure 3E) two-segmented; proximal segment stout, large, and unarmed; second segment bearing small spine at mid-length and distal process fringed with several denticles arranged in row at outer side. Maxilliped (Figure 3F) five-segmented; first segment carrying one inner seta; second segment largest, bearing small spine; third and fourth segments each bearing inner distal seta; final segment slender, terminating in expansion with rake-like structure.

Legs 1 (Figure 5A) and 2 (Figure 5B,C) biramous, consisting of coxa, basis, and two-segmented rami; coxa unarmed; basis bearing one seta on each side, but leg 2 only with outer-side seta; both rami with spines and long plumose setae; armature formula of spines (in roman numerals) and setae (in arabic numerals) as follows:

Leg 3 (Figure 2A) represented by a small process bearing three setae, situated on postero-lateral part of ventral side of prosome. Leg 4 absent.

**Male:** Unknown.

**Remarks:** A total of 37 *Choniomyzon taiwanensis* n. sp. female individuals were found in the external egg mass of a single host longlegged spiny lobster *Panulirus longipes longipes*.

**Etymology**: The new species is the first species of *Choniomyzon* found in Taiwan. The specific name reflects the location of the type-locality.

## 4. Discussion

Only three species (Table 1) have been recognized in the genus *Choniomyzon* to date [4], although several differences are apparent between the descriptions of *C*. *panuliri* provided by Pillai in 1962 [7] and Bradford in 1975 [8]. Morphologically, the new species can be distinguished from its congeners by the combination of the following five character states: (1) small prosome (about 0.84 mm); (2) armature of antennule being 1, 1, 2, 2, 1, 1, 1, 2, 1, 1+1 (aesthetasc), 4, 6+1 (aesthetasc); (3) five-segmented antenna; (4) second segment of antenna bearing one inner seta; (5) two-segmented maxilla. The egg arrangement in an egg sac can also be used as a diagnostic character. The present species has 5–10 eggs in each sac, and the eggs are arranged in a line (Figure 1C and Figure 2B), whereas *C. panuliri* and *C. inflatus* have up to 15 and 14 eggs in a sac, respectively, and they are randomly arranged [6,8]. Another species *C. libiniae* shows a notable difference in egg arrangement, it seems to carry only one egg per sac [10]. In addition to these significant differences, other minor differences shown in Table 2 provide an identification guide to distinguish the species of *Choniomyzon*.

It is suggested that the different species of *Choniomyzon* may select a variety of decapods as hosts [6]. Indeed, based on our finding, the host of *C*. *taiwanensis* n. sp. is different from that of its three congeners (Table 1). It was discovered from examining the external egg mass of the spiny lobster *P*. *longipes longipes*. As pointed out above, several differences are recognized between the descriptions of *C*. *panuliri* provided by Pillai in 1962 [7] and Bradford in 1975 [8]. In addition, they have been found on the different species of spiny lobster hosts. Considering that the species of *Choniomyzon* show high host specificity, the two species examined by Pillai and Bradford [7,8] might be different species. Future study may clarify the true identity of those species, then discuss the potential of using definitive host species for species identification of *Choniomyzon*.

In general, the similar oval-shaped prosome and long caudal rami of the female *Choniomyzon* appear to be mimicking the eggs and egg attachment filaments of their decapod hosts. The egg-oriented mimicry of *Choniomyzon* is suggested to reduce the ejection by the host female during egg grooming [6]. The two parasitic copepods (*C. panuliri* and *C*. *taiwanensis* n. sp.) share the greater number of common characteristics compared with the other two species (e.g., number of segments on the antenna and shape of prosome) (Table 2), and their *Panulirus* hosts (*P*. *homarus*, *P*. *versicolor*, and *P*. *longipes*) are phylogenetically close to each other. Several characteristics (e.g., prosome without wing-like protruding folds and urosome unoverlapped by the prosome) found in the former two species are also present in *C. inflatus* (Table 2), and its host lobster (*I. novemdentatus*) belongs to the same infraorder (Achelata) as *Panulirus*. On the other hand, *C. libiniae* (parasitic on a crab, not on a lobster) shows some unique characteristics (e.g., the final segment of the maxilla bearing with four spiny protuberances, prosome with wing-like protruding folds, and urosome overlapped by the prosome, Table 2). In an attempt of DNA barcoding analysis, we extracted DNA from the copepods for polymerase chain reaction. Amplifications of mitochondrial cytochrome c oxidase subunit I (COI) and 18S ribosomal RNA (18S rRNA) sequences were performed using the universal primers LCO1490 and HCO2198 [12] and SR1 and SR12 [13]. However, the amplification reactions appeared to be unsuccessful, and it indicates that further primer design and analysis are required when more fresh specimens and efficient biomarkers are available. A detailed comparative morphology and molecular phylogenetic analysis among the species of *Choniomyzon* may shed light on the evolutionary process and cosmopolitan distribution of these egg-mimicking copepods.

## 5. Conclusions

Based on the evidence of distinctively morphological features and host preference, *C*. *taiwanensis* n. sp. is confirmed a new species and the first record of *Choniomyzon* species from spiny lobster in Taiwanese waters.

## Figures and Tables

**Table 1:** The information of four *Choniomyzon* copepods parasitic on decapod hosts.

Copepod Species	Host	Distribution	References
*C*. *panuliri*	*P*. *homarus*	India	[7]
	*P*. *versicolor*	British Solomon Islands	[8]
	*Panulirus* spp.	Australia	[2,9]
*C*. *libiniae*	*L*. *spinosa*	Brazil	[10]
*C*. *inflatus*	*I*. *novemdentatus*	Japan	[6]
*C*. *taiwanensis* n. sp.	*P*. *longipes longipes*	Taiwan	This study

**Table 2:** Comparison of morphological features among *Choniomyzon taiwanensis* n. sp. and its congeners (*Choniomyzon panuliri*, *Choniomyzon libiniae*, and *Choniomyzon inflatus*).

	*C*. *panuliri*	*C*. *panuliri*	*C*. *taiwanensis* n. sp.	*C*. *inflatus*	*C*. *libiniae*
Length of prosome (mm)	1.30	1.07	0.84	1.16	0.62–0.83
Antennule	1, 2, 2, 2, 2, 2, 1, 1, 1, 1, 1+1 (aesthetasc), 2+1 (aesthetasc)	1, 1, 2, 2, 1, 1, 1, 2, 1, 1+1 (aesthetasc), 4, 7	1, 1, 2, 2, 1, 1, 1, 2, 1, 1+1 (aesthetasc), 4, 6+1 (aesthetasc)	1, 1, 2, 2, 0, 1, 1, 2, 1, 1+1 (aesthetasc), 3, 4+1 (aesthetasc)	1, 1, 1, 2, 0, 1, 1, 1, 1, 3+1 (aesthetasc), 2, 4+1 (aesthetasc)
No. of segments on the antenna	5	5	5	4	3
Size of terminal spine on the fourth segment of antenna	very long	small	small	incomparable	incomparable
Second segment of antenna	without seta	without seta	with 1 inner seta	without seta	without seta
Distal part of maxillule	with 2 long setae	with 1 spine+3 setae	with 4 setae	with 3 setae	with 4 setae
No. of segments on the maxilla	3	3	2	3	3
Final segment of maxilla	with 1 spine	with 1 spine	with 1 spine	with 1 spine	with 4 spiny protuberances
Maxilliped	1, 3, 2, 1, 1 rake-like structure	1, 1, 1, 1, 1 rake-like structure	1, 1, 1, 1, 1 rake-like structure	1, 1, 1, 1, 1 rake-like structure	1, 1, 1, 1, 1 comb-like structure
A serrate lobe on the distal part of the basis of legs 1 and 2	present	present	absent	absent	absent
Wing-like folds on prosome	absent	absent	absent	absent	present
Urosome overlapped by the prosome	no	no	no	no	yes
Host	*P*. *homarus*	*P*. *versicolor*	*P*. *longipes longipes*	*I*. *novemdentatus*	*L*. *spinosa*

## Data Availability

The data presented in this study are available on request from the corresponding author.
